# A Review of Quantitative and Topical Analysis of Anthocyanins in Food

**DOI:** 10.3390/molecules29081735

**Published:** 2024-04-11

**Authors:** Jorge A. Custodio-Mendoza, Havva Aktaş, Magdalena Zalewska, Jarosław Wyrwisz, Marcin A. Kurek

**Affiliations:** Department of Technique and Food Development, Institute of Human Nutrition Sciences, Warsaw University of Life Sciences (WULS-SGGW), 02-776 Warsaw, Poland; jorge_custodio-mendoza@sggw.edu.pl (J.A.C.-M.); havva_aktas@sggw.edu.pl (H.A.); magdalena_zalewska@sggw.edu.pl (M.Z.); jaroslaw_wyrwisz@sggw.edu.pl (J.W.)

**Keywords:** anthocyanins, quantitative methods, topical methods, analytical chemistry

## Abstract

Anthocyanins, a subclass of flavonoids known for their vibrant colors and health-promoting properties, are pivotal in the nutritional science and food industry. This review article delves into the analytical methodologies for anthocyanin detection and quantification in food matrices, comparing quantitative and topical techniques. Quantitative methods, including High-performance Liquid Chromatography (HPLC) and Mass Spectrometry (MS), offer precise quantification and profiling of individual anthocyanins but require sample destruction, limiting their use in continuous quality control. Topical approaches, such as Near-infrared Spectroscopy (NIR) and hyperspectral imaging, provide rapid, in situ analysis without compromising sample integrity, ideal for on-site food quality assessment. The review highlights the advancements in chromatographic techniques, particularly Ultra-high-performance Liquid Chromatography (UHPLC) coupled with modern detectors, enhancing resolution and speed in anthocyanin analysis. It also emphasizes the growing importance of topical techniques in the food industry for their efficiency and minimal sample preparation. By examining the strengths and limitations of both analytical realms, this article aims to shed light on current challenges and prospective advancements, providing insights into future research directions for improving anthocyanin analysis in foods.

## 1. Introduction

Anthocyanins, a subclass of flavonoids, are vibrant, water-soluble pigments that play a crucial role beyond adding color to fruits and vegetables. Found extensively in red, purple, and blue berries, grapes, apples, plums, cabbage, and other colored grains and vegetables, these compounds enhance the nutritional value of foods. Recognized for their potential health benefits, anthocyanins are being increasingly studied for their ability to prevent and manage chronic diseases such as cardiovascular diseases, obesity, and cancer.

Their concentration varies significantly across different food matrices, with some of the richest sources being berries, red wine, and colored grains like black rice and purple corn. The presence of anthocyanins in these food items not only adds to their aesthetic appeal but also enhances their nutritional profile. One of the most notable properties of anthocyanins is their antioxidant capacity, which plays a critical role in safeguarding health. Research, such as that conducted by Cappellini et al. (2021), has underscored the protective effects of anthocyanins against various chronic diseases, including cardiovascular diseases, obesity, and cancer [1].

Focusing on cardiovascular health, anthocyanins have been observed to exert a positive influence. Epidemiological studies, as noted by Krga & Milenkovic (2019), indicate a correlation between the intake of anthocyanins and a reduced risk of myocardial infarction and mortality related to cardiovascular diseases [2]. Clinical investigations have echoed these findings, showing the beneficial impact of consuming anthocyanin-rich foods on cardiovascular risk markers.

Anthocyanins can induce the activation of phase II enzymes through the antioxidant response element pathway against oxidative stress-induced apoptosis, highlighting their potential to reduce oxidative stress and their chemopreventive potency [3]. These compounds reduce inflammation and improve glucose and lipid metabolism by inhibiting nuclear factor-kappaB activation and increasing PPAR-γ gene expression in metabolic syndrome subjects, which indicates their significant role in modulating metabolic health [4]. Anthocyanins also play a chemopreventive role in atherosclerosis via the activation of Nrf2-ARE as an indicator and modulator of redox, demonstrating their capacity to attenuate inflammation-associated pathogenesis through gene expression modulation [5].

In the realm of neuroprotection, anthocyanins have shown promise in combating neurodegenerative diseases, as reported by Mattioli et al. (2020). Their antioxidant and anti-inflammatory properties are key to this protective role. Additionally, anthocyanins’ ability to modulate gut microbiota further contributes to their neuroprotective effects, underscoring their potential as either complementary or standalone treatment options for various non-communicable diseases, particularly neurodegenerative disorders [6].

Moreover, anthocyanins have been identified as significant players in managing obesity and diabetes. As explored by Sivamaruthi et al. (2020), their intake has been linked to improvements in obesity-related dysbiosis in gut microbiota and a reduction in inflammation in adipose tissue. Furthermore, anthocyanin consumption has been associated with the maintenance or reduction of body weight in obese individuals, as well as with enhancements in metabolism and energy balance [7].

**Figure 1:**
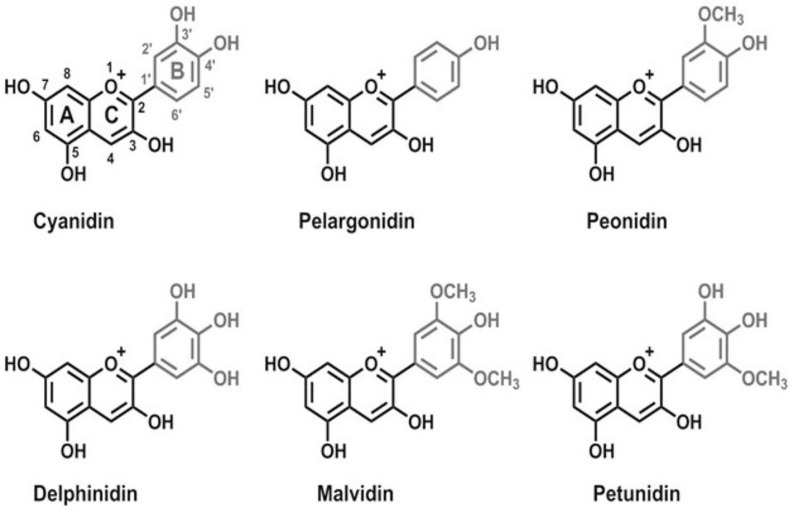
Chemical structures of main anthocyanins: pelargonidin, cyanidin, peonidin, delphinidin, malvidin, and petunidin [12].

Cyanidin, characterized by two hydroxyl groups on the B ring, exhibits a reddish-pink color in acidic conditions, demonstrating the impact of hydroxyl groups on color manifestation. Delphinidin, with three hydroxyl groups, presents a deep blue shade and showcases enhanced metal chelation stability. Malvidin introduces a purple hue through its methoxy group alongside two hydroxyl groups, offering increased stability. Peonidin and petunidin, both featuring a methoxy and two hydroxyl groups, differ in the methoxy position, leading to magenta and blue-violet colors, respectively, and suggest varying degrees of stability and hydrophobicity. Lastly, pelargonidin, with a single hydroxyl group, displays an orange color, indicating a simpler structure that, while vibrant, is less stable than its counterparts (Figure 1) [8]. Factors like pH, temperature, and light exposure affect their stability. This variability necessitates the development of various methods for their stabilization and application in the food industry. Techniques such as acylation and encapsulation have been explored for this purpose, as discussed by Leonarski et al. (2023). These methods are essential for maximizing the use and benefits of anthocyanins in food products, ensuring that their full potential for health promotion is realized [9].

The analysis of anthocyanins is crucial due to their health-promoting properties, including antioxidant, anti-inflammatory, and potential anti-cancer effects. Understanding their bioavailability and stability in foods is essential for maximizing their health benefits and ensuring food quality. Advanced techniques like nanoemulsion and nanoliposome encapsulation are being explored to enhance their stability and bioavailability in food products [10,11].

Fourier Transform Infrared Spectroscopy (FT-IR), Near-infrared Spectroscopy (NIR), and Raman spectroscopy stand as critical analytical tools in food science, each providing distinctive benefits for the swift and non-destructive analysis of food composition. FT-IR and Raman spectra are characteristic of the molecular structure of food components, including anthocyanins, and may be used in conjunction with NMR and MS to elucidate the identification of these compounds [13]. However, these techniques may be used on their own as the basis for rapid topical screening without the need for extensive and time-consuming sample preparation as required for a full quantitative analysis. It detects deviations in the spectroscopic “fingerprint” that indicate an out-of-specification condition as defined by the training dataset. This nuance is crucial and underscores the point that NIR and Raman spectroscopy, while offering rapid analysis, do not directly quantify anthocyanin content. Thus, spectroscopic topical analyses require validation against a quantitative, extractive analytical method with appropriate levels of specificity and accuracy, as defined by various regulatory authorities, to ensure they are fit for purpose when used to screen anthocyanin content in intact food samples [14]. Such methods commonly include liquid chromatography (HPLC or UPLC) with diode array, fluorescence, or mass spectral detectors. The integration of hyperspectral imaging enhances spectroscopic analysis by merging it with digital imaging, allowing for a detailed examination of both the chemical composition and spatial distribution of components such as anthocyanins in heterogeneous food samples. This approach provides a more comprehensive perspective on food quality and composition, reinforcing the importance of a multi-faceted analytical strategy in ensuring food safety and quality [15].

Hyperspectral imaging, not mentioned in the detailed critique but integral to the discussion on topical analytical techniques, further expands on the capabilities offered by spectroscopy by combining it with digital imaging. This technique provides a comprehensive analysis of the chemical composition and spatial distribution of components such as anthocyanins in heterogeneous food samples, offering unparalleled insight into food quality and composition [16].

The integration of machine learning and artificial intelligence with spectroscopic techniques is another innovative approach that enhances the accuracy and efficiency of anthocyanin analysis in food products. This combination of advanced computational methods with traditional spectroscopy is paving the way for more precise and efficient analysis of anthocyanins and other phytochemicals in foods [17].

Furthermore, the development of digital imaging technologies has enabled the visualization and quantification of anthocyanins in food products without causing any damage to the samples. This method is particularly valuable for the food industry, where preserving the integrity of the product during analysis is often crucial [18]. The advancement of these topical methods is revolutionizing the way anthocyanins are analyzed in foods, offering efficient, comprehensive, and practical solutions for food quality and safety assessments.

This review article explores quantitative and topical methods in anthocyanin analysis within food science. Quantitative techniques, such as High-performance Liquid Chromatography (HPLC), Gas Chromatography (GC), and Ultra-performance Liquid Chromatography (UPLC), necessitate altering or consuming the sample for detailed analysis. In contrast, topical methods like Near-infrared (NIR), Fourier Transform Infrared (FTIR), and Raman spectroscopy preserve sample integrity, offering rapid analysis with minimal preparation. This comparison highlights each approach’s benefits and limitations, providing insights into the evolving landscape of anthocyanin analysis and potential future research directions.

## 2. Comparison of Quantitative and Topical Anthocyanin Analyses

In the investigation of anthocyanins in food matrices, the distinction between quantitative and topical analytical techniques is critical in establishing the integrity and utility of results in the context of food quality and health assessments. This section examines the comparative landscape of different techniques, bringing light to their inherent benefits, limits, and implications for the analytical chemistry of food anthocyanins. This approach, although fundamentally changing the sample, provides unparalleled perspectives into the molecular dynamics of foods, notably their anthocyanin content, which is critical for understanding their nutritional and health-promoting qualities.

Anthocyanins in various matrices have been identified and characterized using an array of analytical techniques. High-performance Liquid Chromatography (HPLC) coupled with Mass Spectrometry (MS) has been commonly employed, although MS alone cannot distinguish between positional isomers. Therefore, confirming the identity of anthocyanins often requires complementary methods, such as nuclear magnetic resonance (NMR) spectroscopy or direct comparison with authentic standards to ensure accurate structural elucidation [19]. Chromatographic methods, particularly HPLC, play an important role in the quantitative analysis of anthocyanins. This method’s strength is its capacity to methodically isolate, identify, and measure individual anthocyanins in the middle of complicated dietary matrices. The technique entails dissolving the food sample and allowing it to pass through a column containing a stationary phase. Because anthocyanins interact differently with this phase in the presence of a flowing mobile phase, they are segregated based on their distinct features [20].

Technological breakthroughs have substantially improved the capabilities of chromatographic analysis. Ultra-high-performance Liquid Chromatography (UHPLC), for example, has set new norms for resolution and speed in anthocyanin analysis. When combined with modern detectors such as Diode Array Detection (DAD) and Mass Spectrometry (MS), UHPLC reveals fine data about anthocyanin profiles, adding to our understanding of their functions in food science and nutrition [21,22]. Investigations into the phytochemical profile of butterfly pea flowers via Ultra-performance Liquid Chromatography with Ultraviolet and Mass Spectrometry detection (UPLC/UV-MS) have examined the ionization characteristics of anthocyanins. This study revealed that UPLC/UV-MS, particularly when evaluating both positive and negative ion modes, is a robust technique for identifying and quantifying anthocyanins present in these flowers. In this work, cyanidin derivatives were identified in butterfly pea flower extract, which had not been recognized before. The obtained results demonstrate the potential of butterfly pea flowers for anthocyanin extraction and subsequent usage as a safe colorant in food preparation [23]. The extraction of anthocyanins from food matrices requires numerous stages, which might add unpredictability and significant losses. The problem is to optimize these procedures to ensure anthocyanin purity while guaranteeing effective extraction. This meticulous balance is critical to ensuring the analysis’s representativeness [24]. The diversity of anthocyanin structures present in food samples poses a challenge in standardizing solvent compositions and developing consistent methods for their extraction and analysis.

While quantitative analysis methods are indispensable for the detailed characterization of anthocyanin composition, it is important to consider their environmental impact and ethical considerations regarding material use [25]. Recognizing the essential yet environmentally impactful nature of traditional quantitative analysis methods, there is a growing imperative within the scientific community to explore more sustainable analytical approaches. These include reducing solvent use and developing alternatives that offer a lower environmental footprint. Yet, innovations in chromatography, Mass Spectrometry, and sample preparation are projected to improve their precision, efficiency, and sustainability [26,27].

Topical analysis techniques not only preserve the sample’s integrity for potential future use but also allow for the repeated measurement of samples without altering their physical or chemical state. These approaches are gaining popularity because of their speed, simplicity, and low sample preparation requirements [28]. Spectroscopic methods, particularly Near-infrared (NIR) and Raman Spectroscopy, are at the cutting edge of vibrational topical evaluations. These methods use the interaction of light with the sample to obtain information about its chemical composition, such as the existence and quantity of anthocyanins [29,30]. Topical technologies are especially beneficial for in situ analysis and real-time monitoring of food quality and composition since they provide a non-invasive way to measure anthocyanin levels. While these approaches are less intrusive and more suited to high-throughput analysis, they often have lower specificity, sensitivity, and accuracy than their quantitative counterparts [31].

**Figure 2:**
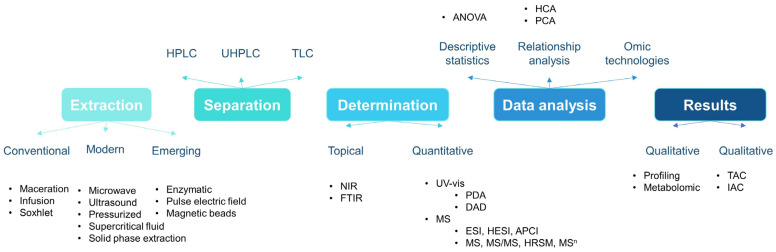
Scheme of the analysis of anthocyanins in foods. HPLC, High-performance Liquid Chromatography; UHPLC, Ultra-high-performance Liquid Chromatography; TLC, Thin-layer Chromatography; NIR, Near-infrared Radiation; FTIR, Fourier Transform Infrared Spectroscopy; UV-vis, Ultraviolet–visible; PDA, photodiode array detector; DAD, diode array detector; ESI, electrospray Ionization; HESI, Heated-ESI; APCI, Atmospheric Pressure Chemical Ionization; MS, Mass Spectrometry; MS/MS, Tandem MS; HRMS, High-resolution MS; Ms^n^, Sequential MS; ANOVA, Analysis of Variance; HCA, Hierarchical Cluster Analysis; PCA, Principal Components Analysis; TAC, Total Anthocyanin Content; IAC, Individual Anthocyanin Content.

Quantitative and topical procedures each serve unique roles in analysis, with their selection depending on the specific aims, sample characteristics, and available resources. Integrating both methods can often provide the most comprehensive understanding (Figure 2). While quantitative methods are critical for precise compositional analysis and the development of food standards, the development of digital imaging technology has substantially improved the capabilities of topical approaches [32]. The combination of these techniques can offer a thorough understanding of anthocyanin content and its implications for food quality and health evaluation.

## 3. Quantitative Anthocyanin Analysis

Assessing the overall antioxidant capacity of food is crucial for health, supporting nutritional profiling, quality control, and informing consumer choices. It also aids researchers in studying health benefits and developing functional foods in response to environmental stressors [7,9,10,33,34]. In this context, analyzing the Total Anthocyanin Content in food can be valuable for quick estimations of antioxidant capacity or comparisons between samples [1,2,6], and examining individual anthocyanin types adds depth to antioxidant capacity estimations and offers numerous insights: understanding bioactivities and providing targeted dietary recommendations [17]; empowering quality control efforts by furnishing a unique “fingerprint” that can detect adulteration and ensure authenticity [35]; preserving the health benefits of anthocyanins during food production and storage by optimizing specific processing and storage conditions [20]; and developing functional foods enriched with specific health-promoting compounds. Finally, research on the specific roles of individual anthocyanins in human health and their potential clinical applications heavily relies on this detailed level of analysis [21].

Chromatographic methods are crucial for determining individual anthocyanins within food matrices, effectively separating complex mixtures based on the physical and chemical properties of their components [22]. Liquid Chromatographic (LC) methods exhibit high sensitivity, selectivity, and resolution, making them well suited for analyzing intricate mixtures such as those found in food and natural products [22,23]. The operation of LC relies on the fundamental principle of separating analytes from a liquid sample through interactions with mobile and stationary phases [36,37,38]. The mobile phase, typically a solvent or a combination of organic and aqueous solutions, carries the sample through the system. Its composition, whether acidified or enriched with salts, depends on separation requirements [9]. The stationary phase, either solid or liquid, is packed into a column or coated onto a support, with varying compositions for different analytes: normal phase for less polar, reverse phase for polar, ion exchange for charged groups, and size exclusion for size-based separation [7]. High-performance Liquid Chromatography (HPLC) and Ultra-high-performance Liquid Chromatography (UHPLC) share the same separation principles based on affinity, size, and electrostatic interactions but differ in pressure and particle size specifications. UHPLC, an advanced form of HPLC, operates at higher pressures (10,000 to 15,000 psi) and uses smaller stationary phase particles, resulting in increased efficiency and faster separations, enhancing resolution and peak capacity, particularly for closely eluting peaks [37,38]. Thin-layer Chromatography (TLC) operates on similar chromatographic principles but uses a thin layer of adsorbent material on a plate as the stationary phase [39]. It is mainly employed for qualitative analysis and preliminary compound separation, offering a quick and straightforward technique suitable for educational purposes. However, TLC tends to be slower than High-performance Liquid Chromatography (HPLC) and Ultra-high-performance Liquid Chromatography (UHPLC) and may not provide the same level of resolution and sensitivity [40]. But TLC gives us the opportunity to test many samples in parallel. The versatility, sensitivity, and high resolution of LC offer unparalleled capabilities for isolating, identifying, and quantifying diverse anthocyanin profiles present in different foods [23]. The detailed analysis of individual anthocyanins plays a vital role in comprehending nutritional content, revealing health benefits, and ensuring food quality control [17,20,21,22,23,41,42]. Ongoing advancements in operative systems, column technologies, optimized gradients, and detection techniques, including topical methods like ultraviolet–visible (UV-Vis) and photodiode array detectors (PDA), as well as quantitative approaches like Mass Spectrometry (MS), further enhance sensitivity, specificity, and provide detailed structural information [23,41,43]. These developments collectively elevate the precision and effectiveness of liquid chromatography in anthocyanin analysis. Table 1 presents a comparison of LC methods developed for the individual determination of anthocyanins in food and natural products from 2020 to the present date.

### 3.1. High-Performance Liquid Chromatography (HPLC)

Colombo et al. (2020) investigated the phenolic composition and yield of the ‘BRS Vitoria’ seedless table grape under varying bunch densities in a South Brazilian vineyard [44]. Their analysis employed solid-phase extraction and a High-performance Liquid Chromatography–diode array detector with tandem mass spectrometry (HPLC–DAD–ESI-MS/MS). The HPLC utilized an Eclipse XDB-C18 column (3.5 μm, 2.1 × 150 mm) from Agilent (Santa Clara, CA, USA), employing a gradient of mobile phases containing acetonitrile (ACN), methanol (MeOH), water, and formic acid (FA) (Table 1). The study successfully identified up to 45 anthocyanins in the seedless grapes. While the phenolic profile remained consistent across densities, the concentrations of specific compounds, such as anthocyanins and flavanols, exhibited variation. The research contributes valuable insights into the phenolic composition of table grapes and the influence of bunch density on yield and phenolic profiles.

Tsiakkas et al. (2020) investigated the impact of factors such as terroir, viticultural practices, vinification methods, and aging conditions on anthocyanins and volatile compounds in wines [45]. The study focused on the indigenous varieties Yiannoudi and Maratheftiko from Cyprus in the vintages 2014, 2015, and 2016. Anthocyanin determination and profiling, involving up to 38 anthocyanins, were conducted using HPLC-DAD-ESI/MS. A Poroshell 120 C18 column (2.7 µm, 50 × 4.6 mm) from Phenomenex (Torrance, CA, USA) and acidified (5% FA) methanol and water as mobile phases were employed for the analysis. The study revealed that, in the initial stages, wines could be readily differentiated by variety, particularly based on anthocyanin composition. However, with aging, differences among samples were influenced by winemaking procedures over time, posing challenges in distinguishing varieties under these conditions.

Zorzi et al. (2020) characterize the polyphenolics and anthocyanins of six edible wild fruits harvested in the north-west of Italy [18]. HPLC-DAD-ESI coupled to High-resolution Mass Spectrometry (HRMS) in positive ion mode was employed for the analysis of 16 anthocyanins employing a Varian Pursuit C18 column (3 μm,150 × 2.0 mm) from Agilent and 0.1% FA in MeOH and water as mobile phases [46].

Kyraleou et al. (2020) determined the phenolic composition of five Greek red grape varieties and utilized the phenolic profile as a tool for varietal discrimination [19]. Ninety grape samples from the years 2017 and 2018 were analyzed, and organic solvents were employed for extraction. The proanthocyanidin profile was determined in both skins and seeds using phloroglucinolysis followed by HPLC-UV and MS detection. Over 13 anthocyanins were identified using HPLC-UV Pinnacle II C18 column (4 μm, 250 mm × 4.6 mm) from Restek (Centre County, PA, USA) and 0.1% FA and MeOH as mobile phases [47].

**Table 1:** High-performance Liquid Chromatography instrumental features for anthocyanin determination (2020–present).

Sample	Flow Rate mL/min	Column	Mobile Phase	Detector	Reference
Seedless grape	0.2	Zorbax Eclipse XDB-C18 column (3.5 μm, 2.1 × 150 mm) Agilent	3:88.5:8.5 (*v*/*v*/*v*) ACN/water/FA (A) 50:41.5:8.5 (*v*/*v*/*v*) ACN/water/FA (B) 90:1.5:8.5 (*v*/*v*/*v*) MeOH/water/FA (C)	ESI-MS/MS	[44]
Wines	2.2	Poroshell 120 C18 (2.7 µm, 4.6 × 50 mm) Phenomenex	95:5 (*v*/*v*) water/FA (A) 95:5 (*v*/*v*) MeOH/FA (A)	DAD-QqQ-MS/MS	[45]
Edible wild fruits	0.2	Varian Pursuit C18 (3 μm,150 × 2.0 mm) Agilent	0.1% FA in MeOH (A), in water (B)	DAD-ESI-HRMS	[46]
Red grape	1	Pinnacle II C18 (4 μm, 250 mm × 4.6 mm) Restek	0.1% FA (A), MeOH (B)	UV-vis	[47]
Dry red wines	0.2	Poroshell 120 EC-C18 core–shell (2.7 μm, 2.1 × 150 mm) Agilent	0.1% FA in water (A), in 1:1 MeOH-ACN (B)	ESI-QqQ-MS/MS	[48]
Wine	0.3	Poroshell 120 EC-C18 (2.7 μm, 2.1 × 150 mm) Agilent	0.1% FA in water (A), in 1:1 MeOH-ACN (B)	QqQ-MS/MS	[49]
Bilberry	1	Hedera ODS-2 C18 (5 µm, 4.6 mm × 250 mm) Nacilai Tesque Inc.	85% FA (A), 22.5:22.5:8.5:41.5 (*v*/*v/v/v*) ACN/MeOH/FA/H_2_O (B)	UV	[35]
Soybean	0.8	C18 core–shell (5 μm, 250 mm × 4.6 mm)	18:82 0.4% TFA in ACN-0.4% TFC in water *	DAD	[50]
Raspberry wine residues	2	Zorbax Eclipse XDB-C18 (5 μm, 4.6 mm × 150 mm) Agilent	5% FA (A), 1% FA in ACN (B)	DAD-MS/MS	[51]
Black chokeberry	1	Zorbax Eclipse XDB-C18 (5 µm, 4.6 × 150 mm) Agilent	2.5% AA (A), ACN (B)	PDA-ESI-MS	[52]
Berries	0.8	Sunfire-C18 (5 µm, 4.6 × 250 mm) Waters	0.1% FA in 10% ACN (A) and 90% ACN (B)	DAD-ESI-QTOF-MS	[53]
Blueberry	1	Zorbax Stablebond SB-C18 (4.6 µm, 250 × 5 mm) Agilent	ACN (A), 0.3% phosphoric acid (B)	UV	[54]
Cranberry	2	ZORBAX Eclipse XDB-C18 (5 μm, 4.6 mm × 150 mm) Agilent	5% FA (A), 1% FA in ACN (B)	UV-MS/MS	[55]
Blackcurrant	0.8	Ascentis Express C18 (2.7 µm, 150 × 4.6 mm) Supelco	2% FA (A), MeOH (B)	UV	[32]
Purple corn	NR	Discovery HS C18 (5 µm, 250 × 4.6 mm) Supelco	0.2% FA in water (A), in 69% MeOH (B)	UV	[56]
Purple carrots	NR	UFLC Aqueous C18 (3 μm, 2.1 × 150 mm) RESTEK	1% FA (A), MeOH (B)	UV-ESI-MS/MS	[57]
Blueberry and strawberry	0.8	Synergi Polar–RP C18 (4 µm, 4.6 × 250 mm) Phenomenex	0.1% FA in water (A), MeOH (B)	ESI-MS/MS	[58]
Grape skins	2.3	Nucleosil 100-5 C18 (5 µm, 4.6 × 250 mm) Macherey–Nagel	5% FA (A), MeOH (B)	DAD	[59]
Roselle	0.8	Zorbax Eclipse plus^®^ C18 (5 μm, 4.6 × 250 mm) Agilent	0.2% FA (A), ACN (B)	UV	[60]
Açaí	2	XBridge BEH C18 (3.5 μm, 4.6 × 50 mm) Waters	2.5% AA in water (A), in ACN (B)	PDA	[61]
Black grape skin and blackberries	1	Zorbax 300 Extended-C18 (5 µm, 4.6 × 150 mm) Agilent	1% FA in water (A), in 80% ACN (B)	DAD-QTOF-MS	[62]
Black beans	1	Intertsil^®^ ODS-3 (5 µm, 4.6 × 250 mm) CPS Analitica	2.5% AA (A), ACN (B)	UV	[63]
Red cabbage, sweet potato, and Tradescantia pallida	0.3	Kinetex C18 (2.6 µm, 4.6 × 150 mm) Phenomenex	10% FA (A), MeOH (B)	DAD-ESI-QTOF-MS/MS	[64]
Colored potato tubers	0.35	Acquity HPLC HSS T3 C18 (1.8 µm, 2.1 × 100 mm) Waters	0.04% AA in water (A), in ACN (B)	MS	[65]
Black rice	0.8	XBridge BEH C18 (5 μm, 4.6 × 50 mm) Waters	0.1% FA in ACN (A), in 5:95 ACN-water (B)	PDA	[66]
Blackcurrant Pomace	1	Luna C18 (5 µm, 250 × 4.6 mm) Phenomenex	50:35:415 FA-ACN-water *	UV	[67]
*Gynura bicolor* DC	0.8	Ultimate AQ C18 (5 μm, 4.6 × 250 mm) Welch Technologies.	1% FA (A), ACN (B)	HRMS	[68]

NR, non-reported; *, isocratic; AA, acetic acid; DAD, diode array detector; FA, formic acid; HRMS, High-resolution Mass Spectrometry; IMS, ion mobility; MS, Mass Spectrometry; MS/MS, tandem mass spectrometry; MS^n^, sequential mass spectrometry; PDA, photodiode array detector; PESI, probe electrospray ionization; QqQ, triple quadrupole; QTOF, quadrupole time of flight; TFA, trifluoroacetic acid; ESI, electrospray ionization.

Zhang et al. (2020) developed a targeted metabolomic method to profile thirty-seven malvidin-derived anthocyanin derivatives in red wine [69]. The methodology involved HPLC tandem ion trap and triple-quadrupole (QqQ) MS, utilizing a Poroshell 120 EC-C18 core–shell column (2.7 μm, 150 mm × 2.1 mm) from Agilent with 0.1% formic acid (FA) in water and a 1:1 mixture of MeOH and ACN as mobile phases. The method demonstrated excellent analytical features, including linearity, sensitivity, determination limits, and repeatability. Later, Zhang et al. (2021) utilized this method to investigate the dynamics of anthocyanin derivatives during wine aging and their correlation with chromatic characteristics [70]. The study employed K-means cluster analysis and partial least squares regression (PLSR) analysis, offering predictive models for assessing the aging process in red wines. This research contributes to understanding the intricacies of anthocyanin evolution during wine aging and provides valuable tools for predicting wine aging outcomes.

Liu et al. (2020) developed and validated a straightforward and expeditious HPLC-UV fingerprint method for the identification of bilberry extract [10]. The study employed a Hedera ODS-2 C18 column (5 µm, 4.6 mm × 250 mm) from Nacilai Tesque Inc. (Kyoto, Japan) and utilized an 85% formic acid (FA) solution and a 22.5:22.5:8.5:41.5 (*v*/*v*/*v*/*v*) ACN/MeOH/FA/H_2_O solution as mobile phases. Six batches of bilberry extract obtained from different manufacturers were utilized to establish the HPLC fingerprint. This method serves as a valuable tool for ensuring the authenticity and quality control of bilberry products in the market [35].

Krishnan et al. (2020) employed a modified HPLC-DAD method for anthocyanin fingerprinting in differentially pigmented exotic soybean genotypes [50]. The elution profile of anthocyanins, including cyanidin-3-glucoside, delphinidin-3-glucoside, and petunidin-3-glucoside, was determined using a C18 core–shell column (5 μm, 250 mm × 4.6 mm) with an isocratic flow of 18:82 ACN-water, both containing 0.4% trifluoroacetic acid. Positive correlations were observed among various variables, including monomeric anthocyanin content, as revealed by clustering and heat map analysis. This HPLC-UV method provides valuable insights into anthocyanin composition in soybean genotypes, offering a tool for further research on germplasm evaluation and the development of nutritionally enriched varieties.

Xue et al. (2021) optimized the extraction conditions for anthocyanins from raspberry wine residues using ultrasound-assisted enzymatic extraction [51]. The main anthocyanins, cyanidin-3-glucoside and cyanidin-3-rutinoside, were identified through HPLC-DAD tandem mass spectrometry (MS/MS) and nuclear magnetic resonance (NMR). Chromatographic separation was achieved using a Zorbax Eclipse XDB-C18 column (5 μm, 4.6 mm × 150 mm) from Agilent, employing a gradient of mobile phases comprising 5% FA and 1% FA in ACN.

Zhu et al. (2021) identified anthocyanins present in *Aronia melanocarpa* using mass spectrometric features [52]. The anthocyanins of *A. melanocarpa* were analyzed through UV-Vis, HPLC-DAD, and LC-EIS/MS methods. Four significant anthocyanins were identified, namely cyanidin-3-galactoside, cyanidin-3-arabinoside, cyanidin-3-glucoside, and cyanidin-3-xyloside. Chromatographic separation was performed using a Zorbax Eclipse XDB-C18 column (5 µm, 4.6 × 150 mm) from Agilent with a gradient of 2.5% acetic acid (AA) and ACN as mobile phases. The methodology employed in this study provides an effective tool for anthocyanin identification, and the results suggest that anthocyanins from *A. melanocarpa* exhibit potent antioxidant activity, which could be crucial for future research on chokeberry-based functional food products.

Kim et al. (2021) employed HPLC-DAD and quadrupole time-of-flight QTOF-MS to identify individual phenolic compounds, including 5 anthocyanins, in Aronia [23]. The chromatographic separation utilized a Sunfire-C18 column (5 µm, 4.6 × 250 mm) from Waters (Milford, MA, USA), with 0.1% FA in 10% ACN and 90% ACN as mobile phases. Their study revealed variations in the phytochemical composition among different berries. Aronia and Korean raspberry exhibited the highest levels of total phenol content (TPC), total flavonoid content (TFC), and Total Anthocyanin Content [53]. Conversely, red raspberry demonstrated the highest ascorbic acid content. These findings provide valuable insights into the nutritional composition of various berries and their potential health benefits.

Zhou et al. (2021) optimized the chromatographic conditions of High-performance Liquid Chromatography (HPLC) to investigate six major anthocyanins (delphinidin-3-O-glucoside, cyanidin-3-O-glucoside, petunidin-3-O-glucoside, pelargonidin-3-O-glucoside, peonidin-3-O-glucoside, and malvidin-3-O-glucoside) in blueberry [54]. The optimized separation conditions were applied in both analytical and semi-preparative HPLC [24]. The results demonstrated effective separation of the six anthocyanins using acetonitrile–water (containing 0.3% phosphoric acid) as the mobile phase with gradient elution and a Zorbax Stablebond SB-C18 (4.6 µm, 250 × 5 mm) from Agilent at a detection wavelength of 520 nm. Moreover, positive correlations between antioxidant activities and the levels of phenolic compounds and anthocyanins suggest that these compounds contribute to the antioxidant potential of the berry samples.

Xue et al. (2021) optimized the ultrasound-assisted extraction (UAE) process for anthocyanins from cranberry, achieving the highest yield (7.25 mg/g) through specific parameters [51]. They employed HPLC-UV-MS/MS, using a ZORBAX Eclipse XDB-C18 column (5 μm, 4.6 mm × 150 mm) from Agilent and 5% FA and 1% FA in ACN to identify seven anthocyanins, including novel ones like peonidin-3-(6-malon)-glucoside and pelargonidin-3-(6-malon)-glucoside. The UAE method proved effective, indicating its potential for extracting bioactive compounds from cranberry.

Šimerdová et al. (2021) developed a HPLC-DAD method for the rapid separation and determination of anthocyanins in fifteen blackcurrant cultivars [32]. The method employed 2% FA and MeOH as mobile phases and an Ascentis Express C18 column (2.7 µm, 150 × 4.6 mm) from Supelco (Bellefonte, PA, USA). The individual anthocyanin proportions were specific to each cultivar, and a consistent profile was observed over a three-year period for eight cultivars. Cultivars with the highest average Total Anthocyanin Content were ‘Ben Gairn’, ‘Ceres’, and ‘Ometa’.

Barba et al. (2022) studied the efficiency of infrared (IR)-assisted extraction compared with the traditional water bath method and optimized conditions using Response Surface Methodology [56]. HPLC-UV, utilizing a Discovery HS C18 column (5 µm, 250 × 4.6 mm) from Supelco and a mobile phase of 0.2% formic acid in water and 69% MeOH, was employed to identify two anthocyanins and other polyphenols. The extracted material from purple corn cobs is proposed as a natural colorant for pickled turnips, providing a healthier alternative to the banned carcinogenic synthetic dye, rhodamine B.

Perez et al. (2022) used HPLC- UV-ESI-MS/MS with 1% FA and MeOH and a UFLC Aqueous C18 column (3 μm, 2.1 × 150 mm) from Restek to study the potential of carrot anthocyanins as food colorants and nutraceuticals by assessing their physicochemical stability and antioxidant capacity [28]. The study suggests that the higher stability of carrot colorants is attributed to their abundance in acylated anthocyanins emphasizing their potential as natural and stable alternatives to synthetic and other natural colorants [57].

Mustafa et al. (2022) developed a HPLC-MS/MS method for simultaneously determining 36 phenolic compounds, including 7 anthocyanins, in blueberry and strawberry, and their fruit jam [58]. They used a Synergy Polar–RP C18 column (4 µm, 4.6 × 250 mm) from Phenomenex and 0.1% FA and MeOH as mobile phases. The developed HPLC-MS/MS method is deemed valuable and reliable for quality control and authentication analyses of blueberry and strawberry fruits, as well as their commercial food products, such as jams.

Kalogiouri et al. (2022) developed a rapid and simple HPLC-DAD method for determining anthocyanins in three red Greek wine grape varieties (Kotsifali, Limnio, and Vradiano) [30]. The separation was carried out using a Nucleosil 100-5 C18 column (5 µm, 4.6 × 250 mm) from Macherey–Nagel (Düren, Germany) and a 5% FA and MeOH mobile phases. The method was used to analyze sixteen samples from the main regions of each grape variety and official ampelographic collections in Greece during the 2020 growing season [59]. Hierarchical Cluster Analysis (HCA) revealed notable differences in anthocyanin content among the cultivars.

Khan et al. (2022) developed an HPLC-UV method for quantifying 5 anthocyanins in spray-dried aqueous extract (SDE), oral powder, and compressible lozenge formulations [60]. Chromatographic conditions using Eclipse plus^®^ C18 column from Agilent, mobile phase (water with 0.2% FA and ACN), and detection at 525 nm were optimized. Forced degradation studies were conducted under different conditions on the pure compound, extract, and developed formulations. The study ensured the method’s reliability and suitability for stability assessment and quantification of anthocyanin content in the specified formulations.

Maciel-Silva et al. (2022) aimed to extract anthocyanins from dried and semi-defatted açaí pulp using environmentally friendly methods [61]. The approach involved pressurized liquid extraction (PLE) coupled with in-line purification through solid-phase extraction (SPE) and online analysis using HPLC-PDA. Critical parameters were optimized through Response Surface Methodology (RSM). PLE, conducted with acidified water at varying pH and temperatures, and SPE, optimized for anthocyanin elution with ethanol, were integrated into a powerful system. The coupled PLE-SPE × HPLC-PDA used an Xbridge BEH C18 from Waters (3.5 μm, 4.6 × 50 mm) and 2.5% AA in water and ACN as mobile phases. The system produced a highly concentrated anthocyanin extract, demonstrating its effectiveness for real-time monitoring of the extraction process.

Paun, Botoran, and Niculescu (2022) used HPLC-DAD-QTOF-MS to identify and quantify anthocyanins in black grape skins and blackberries while also determining Total Phenolic Content and antioxidant capacity [62]. Extracts were prepared using an ultrasound-assisted acidified ethanol and methanol method, with 80% methanol proving to be the most effective. HPLC-DAD-MS analysis carried out using a Zorbax 300 Extended-C18 column from Agilent (5 µm, 4.6 × 150 mm) and 1% FA in water and 80% ACN revealed five glycosylated anthocyanins in blackberries and eight in grape skins, including seven anthocyanin derivatives.

Melini et al. (2023) optimized the ultrasound-assisted extraction of phenolic compounds, including two anthocyanins, from black beans using Response Surface Methodology (RSM) [63]. Phenolic compounds are valued for their biological activities and potential health benefits. The researchers used a Generally Recognized Safe solvent (ethanol) to maximize Total Anthocyanin Content (TAC) and Total Phenolic Content (TPC). They utilized the HPLC-UV method, employing an Intertsil^®^ ODS-3 column (5 µm, 4.6 × 250 mm) (GL Sciences, Nishi Shinjuku, Japan) and a mobile phase consisting of 2.5% AA and ACN to identify cyanidin-3-O-glucoside and peonidin-3-O-glucoside, along with phenolic acids present in the extracts.

Steingass et al. (2023) characterized 18 anthocyanins in red cabbage, sweet potato, and Tradescantia pallida leaves using HPLC-DAD-MS/MS and MS^n^ [64]. The chromatographic separation employed a Kinetex C18 column (2.6 µm, 4.6 × 150 mm) from Phenomenex with a mobile phase gradient consisting of 10% formic acid (FA) and methanol (MeOH). Their findings revealed that acylated anthocyanins demonstrate superior thermal stability during heating compared to a commercial Hibiscus-based food dye, with Tradescantia extract exhibiting the highest stability.

Qi et al. (2023) used HPLC-MS to determine the anthocyanins of colored potato tubers grown at different altitudes using an Acquity HPLC HSS T3 C18 column from Waters (1.8 µm, 2.1 × 100 mm) and 0.04% AA in water and in ACN [65]. The main anthocyanins found in purple clones were malvidin, petunidin, and cyanidin.

Mackon et al. (2023) combined HPLC-PDA and transcriptome analysis to profile the changes in anthocyanin content and gene expression dynamics at three developmental stages (milky, doughy, and mature) of black rice [66]. Unique gene expressions were identified in milky, doughy, and mature stages. Significant gene changes were observed in the milky vs. mature stages. Key anthocyanin biosynthesis genes were highly expressed at the dough stage, contributing to the observed peak.

Kruszewski & Boselli (2024) utilized HPLC-PDA to investigate the impact of ultrasound-assisted extraction (UAE) parameters on anthocyanin content and color parameters in blackcurrant pomace extracts [67]. The anthocyanin-rich pomace underwent extraction with varying temperatures, times, and material/solvent ratios, using two solvents: water–ethanol acidified with HCl, and water acidified with citric acid. Chromatographic separation employed a Luna C18 column (5 µm, 250 × 4.6 mm) from Phenomenex with a mobile phase of 50:35:415 FA-ACN-water in isocratic mode. The extracts, particularly those from the water–citric acid solvent, were recognized as potent colorants suitable for applications like food coloring. Ultrasonic extraction demonstrated success in obtaining pigments from blackcurrant pomace.

Jia et al. (2024) characterized anthocyanins in *Gynura bicolor* using HPLC-Q-Orbitrap MS. The study identified five anthocyanin compounds, with delphinidin 3-O-neohesperidoside being the predominant one. Chromatographic separation utilized an Ultimate AQ C18 column (5 μm, 4.6 × 250 mm) from Welch (West Haven, CT, USA) (with 1% formic acid (FA) and acetonitrile (ACN) as mobile phases. Microwave-vacuum drying (MVD) was employed, demonstrating an 89% reduction in drying time compared to hot air drying (HAD). The findings suggest that MVD is an effective method for producing high-quality dried *G. bicolor* products with significantly reduced processing times compared to HAD [68].

From 2020 to 2024, HPLC has been extensively utilized for anthocyanin analysis across diverse plant materials, as highlighted in various studies. These studies showcase the diverse applications of HPLC in anthocyanin research, encompassing the evaluation of the impact of processing and storage on anthocyanin content, ensuring the authenticity and consistency of commercial products, distinguishing between different plant varieties, identifying potential sources of bioactive compounds, developing efficient methods for extracting anthocyanins, and studying the degradation behavior of anthocyanins under different conditions, among other applications.

Each study employs a specific HPLC setup, though there is a consensus on using C18 columns in reverse mode with particle sizes ranging between 1.8 and 5 μm, internal diameters between 2 and 4.6 mm, and lengths between 50 and 250 mm. Novel C18-based packaging technologies have been introduced, including core–shell particles offering higher efficiency and faster analysis compared to fully porous particles [50,69], C18 chain structures [62], and Synergi Polar-RP C18 from Phenomenex with an embedded polar group for enhanced selectivity [58]. Additionally, BEH particles, formed by combining two different silica molecules, and HSST3 C18, combining high-strength silica with a C18-bonded phase, showcase enhanced mechanical strength and chemical stability [61,65,66].

Most authors opted for a mobile phase gradient comprising acetonitrile and/or methanol, along with water acidified with 0.1–10% formic acid, although trifluoroacetic and acetic acid have also been utilized. Notably, Kruszewski et al. employed an isocratic mode with a mobile phase composition of 50:35:415% (*v*/*v*/*v*) formic acid–acetonitrile–water [67].

Regarding detection methods tailored to specific analytes and research goals, UV-Vis techniques, including basic and advanced (PDA, DAD), as well as MS spectrometry, including single quadrupole, tandem MS/MS, and High-resolution Mass Spectrometry (HRMS), have been employed. The selection of detection systems is guided by the required level of specificity and sensitivity, information needed for identification or characterization, and available resources and budget constraints. For complex analyses necessitating both high specificity and detailed structural information, hyphenated techniques such as DAD-MS/MS, DAD-ESI-HRMS, or DAD-ESI-QTOF-MS prove to be powerful tools.

### 3.2. Ultra-High-Performance Liquid Chromatography (UHPLC)

UHPLC has been extensively used for the individual determination of anthocyanins in the last five years, mostly employing C18 columns with varying particle sizes, lengths, and internal diameters (Table 2). The Acquity UPLC BEH C18 column (1.7 µm; 2.1 × 150 mm) from Waters has been utilized in different applications. The BEH C18 stationary phase is a hybrid silica-based particle with a bonded C18 phase with a pH of 1–12 and a maximum pressure of 18,000 psi. It was employed with a mobile phase of 0.1% FA in water and in ACN by Qu et al. (2020) in their metabolic profiling of yellow- and black-seeded rapeseed, identifying 12 anthocyanins [40]. Sabino et al. (2021) used the same column to determine 3 anthocyanins from Jambolan fruit using pressurized liquid extraction and ultrasound methods [71]. Wang et al. (2022) applied it to determine 12 anthocyanins in orange and yellow petal rapeseed [72].

Using the same column and 2% FA and ACN (or ACN-MeOH 1:1) has been employed for determining 9 anthocyanins in pomegranate peel by Man et al. (2022) [73] and 30 anthocyanins in blueberry by Wang et al. (2022) [74]. The same column and 5% FA and CAN were utilized in the determination of 9 anthocyanins in Rhododendron arboretum by Bhatt et al. (2022) [75]. This same column, with acidification of both organic and aqueous mobile phases using 1% FA, was used for the determination of 9 anthocyanins in corn kernels by Hong, Netzel, and O’Hare (2020) [76]. The use of trifluoroacetic acid was also proposed using this same column by Tsamo et al. (2020) in their seed coat metabolite profiling of cowpeas [77].

The shorter length of the Acquity UPLC BEH C18 (1.7 μm, 2.1 mm) has also been explored for the determination of anthocyanins. The 100 mm length version was utilized by D’Angelo et al. (2020) in characterizing Tupy, Guarani, and Xavante blackberry cultivars, determining 4 anthocyanins using 0.01% FA and MeOH as mobile phases [78]. Salem et al. (2020) employed the same version with 0.1% FA and 100% ACN (or MeOH) in their metabolic profiling of Hibiscus sabdariffa, identifying 4 anthocyanins [79]. Huang et al. (2021) used it in their metabolomic profiling of *Kadsura cossinea* fruits, identifying 17 anthocyanins [80], and Ha et al. (2021) identified 10 anthocyanins in black seed-coated Korean adzuki beans with 0.1% FA and 100% ACN (or MeOH) [74].

After testing different columns, the 100 mm length ACQUITY UPLC BEH C18 (1.7 μm, 2.1 × 100 mm) with 1% FA and ACN was used by Li et al. (2022) to determine 10 anthocyanins in grapes [81]. Stój, Kapusta, and Domagała (2020) utilized the same column and 2% FA in both aqueous solution and ACN to identify 24 anthocyanins in red wines and classify them based on this [53]. Guo et al. (2020) used 0.04% AA with this column for determining 11 anthocyanins in nectarine and peach [82], and Sendri et al. (2022) used 0.3% phosphoric acid and ACN to determine 8 anthocyanins in Ipomoea nil [83]. Most recently, the shorter 50 mm version was employed with 0.5% FA and ACN by Li, Simon, and Wu (2024) for the determination of Hibiscus sabdariffa products [84]. Notably, shorter columns lead to faster analysis times and sharper peaks due to reduced diffusion times and optimized flow rates. The ACQUITY UPLC BEH C18 (5 µm; 2.1 × 50 mm) with 2% FA in both aqueous and organic phases was used by Canedo-Reis et al. for the quantification of 4 anthocyanins in different grape varieties’ juice [85].

The Acquity UPLC HSS T3 C18 (1.8 µm, 2.1 mm × 100 mm) from Waters, a trifunctional C18-bonded silica gel core phase with a pH range from 2 to 8 and a maximum pressure of 15,000 psi, was utilized by Lai et al. (2020) to determine 15 anthocyanins in Liquidambar formosana ‘Nanlinhong’ trees and study their seasonal variations [60]. Additionally, Zheng et al. (2023) employed the same column in their metabolic profiling of grape skins using 0.1% FA and CAN [86].

Zheng et al. (2020) utilized a ZORBAX XDB-Phenyl column (5 μm, 4.6 × 250 mm) from Agilent, containing a dimethylphenylethylsilane phase with a pH range of 2–11 and a maximum pressure of 600 psi. They used 0.1% FA in both organic and aqueous phases to determine 9 anthocyanins in Perillae Folium [62]. Yue et al. (2024) employed the same mobile phase with a Zorbax 300 Extended C18 column (5 µm, 4.6 × 150 mm), featuring an extra dense bonding C18 phase with a pH range of 2–12 and a maximum pressure of 6000 psi, for the determination of 6 anthocyanins in pomegranate pomace [87]. Zhang et al. (2021) performed ultrasound-assisted extraction of 6 anthocyanins from Tibetan hulless barley using a ZORBAX Eclipse Plus column (1.8 μm, 4.6 × 100 mm), which is an enhanced C18-bonded silica phase that supports up to 15,000 psi of pressure, with a pH range of 1–12. They used 0.1% trifluoroacetic acid in water and CAN [88]. Thuy et al. (2021) used a Traditional C18-bonded silica column (pH range: 2–11, maximum pressure: 6000 psi) for the determination of 6 anthocyanins from butterfly pea flowers [23]. Xu et al. (2022) employed a ZORBAX RRHD SB-C18 column (1.8 µm, 2.1 × 50 mm), a fully porous C18 phase with a pH range of 1–12 and a maximum pressure of 15,000 psi, for the determination and monitoring of 10 anthocyanins in Sugarcane [89].

The UPLC SB-C18 column (1.8 µm, 2.1 mm × 100 mm) from Agilent was used by Dai et al. in the determination of 4 anthocyanins from *Rhododendron liliiflorum* [90] and more recently by Zeng et al. (2023) in their characterization of 104 anthocyanins from rapeseed petals [91]. Both studies used 0.1% FA in water and ACN as mobile phases, and this C18-bonded silica column has a pH range of 2–8 and is capable of supporting up to 17,000 psi of pressure.

Phenomenex’s Gemini C18 column with different particle sizes and lengths has also been recently used. Li, Inbaraj, and Chen (2023) employed the 5 µm, 4.6 × 250 mm column to determine 2 anthocyanins in mangosteen peel by HRSM using 2% FA in water and MeOH [92]. Medic et al. (2023) used the 3 µm, 4.6 × 150 mm column to determine 11 anthocyanins in bilberry and blueberry liqueurs using 0.1% FA in 3% ACN and 97% CAN as mobile phases [93].

Cioni et al. (2023) utilized a Kinetex Biphenyl C-18 (2.6 µm, 2.1 × 100 mm) column from Phenomenex for the determination of 7 anthocyanins from prunus fruit using 0.1% FA in water and MeOH [94]. This column combines traditional C18 functionality with biphenyl groups, offering additional π-π interactions and dipole–dipole interactions, leading to enhanced selectivity for various compounds compared to standard C18 columns. Remarkably, de Souza Mesquita et al. (2023) developed a universal method to determine up to 53 anthocyanins in black grape, blueberry, blackberry, strawberry, pomegranate, Brazilian berry, eggplant, red onion, and red cabbage using a Kinetex C18 (2.6 µm, 4.6 × 100 mm) column and, after testing different conditions, 0.25 M citric acid and ethanol as mobile phases [95].

Wen et al. (2021) employed both HPLC-UV and UHPLC-QTOF-MS in their determination of 4 anthocyanins from black chokeberry [96]. HPLC-UV determination was carried out using an Inertsil ODS-SP C18 (5 µm, 4.6 × 250 mm) column with 10% AA and 1% phosphoric acid in water and ACN as mobile phases, while UHPLC-MS identification was conducted using an HSS T3 column (2.1 mm × 100 mm, 1.8 µm) with 0.2% FA in water and ACN as mobile phases.

UHPLC systems commonly support higher pressures, allowing the use of short columns with smaller particle sizes, significantly reducing analysis time. From 2020 to 2024, most authors used C18 columns with similar dimensions as in HPLC applications, and also with particle sizes between 1.7 and 5 μm. Zheng et al. and Cioni et al. employed chromatography columns with mixed-mode stationary phases that combine C18 and phenyl functionalities, incorporating balanced hydrophobic and π-π interactions for separating a wider range of analytes compared to pure C18 [91,94]. Similarly, mobile phases encompass combinations of acetonitrile, methanol, and water, which are acidified with formic, acetic, and trichloroacetic acid. Remarkably, de Souza Mesquita et al. introduced a greener approach by using ethanol as an organic solvent and citric acid to determine up to 53 anthocyanins in fruits and berries using PDA [97]. Regarding detector systems, within UHPLC applications, most authors choose the use of MS/MS, HRMS, or hyphenated UV-MS and UV-MS/MS techniques to identify and quantify anthocyanins in various plant materials, including various fruits, seeds, flowers, and plants.

**Table 2:** Ultra-high-performance Liquid Chromatography instrumental features for anthocyanin determination (2020–present).

Sample	Flow Rate mL/min	Column	Mobile Phase	Detector	Reference
Rapeseed	0.3	ACQUITY UPLC BEH C18 (1.7 µm; 2.1 × 150 mm) Waters	0.1% FA n water (A), in ACN (B)	HESI-MS/MS	[98]
Jambolan fruit	0.4	ACQUITY UPLC BEH C18 (1.7 µm; 2.1 × 150 mm) Waters	0.1% FA in water (A), in ACN (B)	QTOF-MS	[71]
Rapeseed	NR	ACQUITY UPLC BEH C18 (1.7 µm; 2.1 × 150 mm) Waters	0.1% FA in water (A), in ACN (B)	HESI-MS/MS	[72]
Pomegranate peel	0.4	ACQUITY UPLC BEH C18 (1.7 µm; 2.1 × 150 mm) Waters	2% FA (A), ACN (B)	QTOF-MS	[73]
Blueberry	NR	ACQUITY UPLC BEH C18 (1.7 µm; 2.1 × 150 mm) Waters	1:1 MeOH-ACN (A), 2% FA (B)	MS/MS	[49]
Rhododendron arboreum	0.25	ACQUITY UPLC BEH C18 (1.7 µm; 2.1 × 150 mm) Waters	5% FA (A), ACN (B)	UV-ESI-IMS-MS/MS	[75]
Cowpea	NR	ACQUITY UPLC BEH C18 (1.7 µm; 2.1 × 150 mm) Waters	0.1% TFA (A), ACN (B)	PDA-QTOF-MS	[77]
Corn kernels	2	ACQUITY UPLC BEH C18 (1.7 µm; 2.1 × 150 mm) Waters	92:7:1 (*v*/*v*/*v*) ACN/water/FA (A) 1% FA in ACN (B)	DAD-ESI-MS/MS	[76]
Black berry	NR	ACQUITY UPLC BEH C18 (1.7 μm, 2.1 × 100 mm) Waters	0.01% FA in water (A), in MeOH (B)	ESI-QTOF-MS/MS	[78]
Malvaceae	0.4	ACQUITY UPLC BEH C18 (1.7 μm, 2.1 × 100 mm) Waters	0.1% FA in water (A), in ACN (B)	HRMS	[79]
Fruits	0.35	ACQUITY UPLC BEH C18 (1.7 μm, 2.1 × 100 mm) Waters	0.1% FA in water (A), in MeOH (B)	ESI-MS/MS	[80]
Black seed-coated adzuki bean	0.3	ACQUITY UPLC BEH C18 (1.7 μm, 2.1 × 100 mm) Waters	0.1% FA in water (A), in ACN (B)	HRMS	[99]
Grapes	0.3	ACQUITY UPLC BEH C18 (1.7 μm, 2.1 × 100 mm) Waters	1% FA (A), ACN (B)	Q-TOF-MS	[85]
Red wines	0.6	ACQUITY UPLC BEH C18 (1.7 μm, 2.1 × 100 mm) Waters	2% FA in water (A), in 40% ACN (B)	PDA-MS/MS	[100]
Nectarine and peach	1	ACQUITY UPLC BEH C18 (1.7 μm, 2.1 × 100 mm) Waters	0.04% AA in water (A), in MeOH (B)	ESI-QTOF-MS	[82]
Ipomoea nil	NR	ACQUITY UPLC BEH C18 (1.7 μm, 2.1 × 100 mm) Waters	0.3% phosphoric acid (A), ACN (B)	ESI-MS/MS	[83]
Hibiscus sabdariffa	0.4	ACQUITY UPLC BEH C18 (1.7 µm, 2.1 × 50 mm) Waters.	0.5% FA (A), ACN (B)	DAD-MS	[84]
Grapes juice	0.45	ACQUITY UPLC BEH C18 (5 µm; 2.1 × 50 mm) Waters	2% FA (A), 90:2:8 (*v*/*v*/*v*) MeOH/FA/water (B)	ESI-MS	[85]
Liquidambar formosana	0.4	ACQUITY UPLC HSS T3 C18 (1.8 µm, 2.1 mm × 100 mm) Waters	0.04% AA in water (A), 0.04% AA in ACN (B)	ESI-MS/MS	[101]
Grape skins	0.35	ACQUITY UPLC HSS T3 C18 (1.8 µm, 2.1 mm × 100 mm) Waters	0.1% FA (A), ACN (B)	ESI-MS/MS	[86]
Perillae Folium	1	ZORBAX XDB-Phenyl (5 μm, 4.6 × 250 mm) Agilent	0.1% FA in ACN (A), in water (B)	ESI-QTOF-MS/MS	[87]
Pomegranate pomances	0.2	Zorbax 300 Extended-C18 (5 µm, 4.6 × 150 mm) Agilent	0.1% FA in water (A), in ACN (B)	HRMS	[88]
Tibetan hulless barley	0.8	ZORBAX eclipse Plus (1.8 μm, 4.6 × 100 mm) Agilent	0.1% FA in water (A), in ACN (B)	QTOF-MS	[102]
Butterfly Pea Flowers	0.3	Zorbax Eclipse C18 (1.8 µm, 2.1 × 50.0 mm,) Agilent	0.1% FA in water (A), in ACN (B)	UV-MS	[23]
Sugarcane	0.2	ZORBAX RRHD SB-C18 (1.8 µm, 2.1 × 50 mm) Agilent	0.1% TFA (A), ACN (B)	ESI-QTOF-MS/MS	[89]
Rhododendron liliiflorum	0.35	UPLC SB-C18 column (1.8 µm, 2.1 mm × 100 mm) Agilent	0.1% FA in water (A), in ACN (B)	ESI-QTrap-MS/MS	[90]
Rapeseed Petals	0.35	UPLC SB-C18 column (1.8 µm, 2.1 mm × 100 mm) Agilent	0.1% FA in water (A), in ACN (B)	HESI-MS/MS	[91]
Mangosteen Peel	0.5	Gemini C18 (5 µm, 4.6 × 250 mm) Phenomenex	2% FA in water (A), in ACN (B)	ESI-HRMS	[92]
Bilberry & Blueberry Liqueurs	NR	Gemini C18 (3 µm, 4.6 × 150 mm) Phenomenex	0.1% FA in 3% ACN (A), in 97% ACN (B)	HESI-MS/MS	[93]
Clitoria ternatea	0.5	Aqua C18 (5 μm, 4.6 × 150 mm) Waters	0.1% TFA (A), ACN (B)	DAD-ESI-MS	[103]
Prunus fruit	0.5	Kinetex Biphenyl C-18 (2.6 µm, 2.1 × 100 mm) Phenomenex	0.1% FA in water (A), in MeOH (B)	DAD-ESI-HRMS	[94]
Black grape, blueberry, blackberry, strawberry, pomegranate, Brazilian berry, eggplant, red onion and red cabbage	0.5	Kinetex C18 (2.6 µm, 4.6 × 100 mm) Phenomenex	0.25 M citric acid (A), EtOH (B)	PDA	[97]
Black chokeberry	1	Intertsil ODS-SP C18 (5 µm, 4.6 × 250 mm) GL Sciences Inc. HSS T3 column (2.1 mm × 100 mm, 1.8 µm).	10% AA + 1% Phosphoric acid in water (A), ACN (B) 0.2% FA in water (A), in ACN (B)	PDA QTOF-MS	[96]

NR, non-reported; AA, acetic acid; DAD, diode array detector; FA, formic acid; HESI, heated electrospray ionization; HRMS, High-resolution Mass Spectrometry; IMS, ion mobility; MS, Mass Spectrometry; MS/MS, tandem mass spectrometry; MS^n^, sequential mass spectrometry; PDA, photodiode array detector; PESI, probe electrospray ionization; QqQ, triple quadrupole; QTOF, quadrupole time of flight; TFA, trifluoroacetic acid; ESI, electrospray ionization.

### 3.3. Thin-Layer Chromatography

Thin-layer Chromatography (TLC) has been a valuable tool in the analysis of anthocyanins, offering simplicity, cost-effectiveness, and rapid results, although it serves as a complementary technique rather than the main player [104]. TLC allows for parallel measurement of many samples rather than singly by (U)HPLC. Newer stationary phases, optimized solvent systems, and derivatization methods have boosted its sensitivity and resolution, but limitations in quantitative analysis and interference from other compounds persist [104,105]. The choice of the mobile phase, often a combination of organic solvents and acids, is crucial for achieving optimal separation [106]. Visualization of anthocyanins on TLC plates is typically facilitated using reagents like aluminum chloride or diphenylboric acid aminoethyl ester, or by exposing the plates to UV light. Densitometry and image analysis software are employed for quantitative analysis, enabling the measurement of spot intensities [107]. The use of image analysis makes TLC semi-quantitative. While TLC has been advantageous for preliminary screening and qualitative analysis in the last five years (Table 3), more advanced techniques such as High-performance Liquid Chromatography (HPLC) and Mass Spectrometry (MS) are frequently utilized for accurate quantification and in-depth profiling of individual anthocyanin compounds (Table 3).

Suganyadevi, Saravanakumar, and Mohandas (2021) used TLC to confirm the isolation of anthocyanins extracted from red sorghum bran using a Silica gel 60 F254 TLC plate (20 × 10 cm) from Merck (Darmstadt, Germany) and ethyl acetate (EtOAc), water, and FA (85:8:6 *v*/*v/v*) as the mobile phase, identifying up to 4 anthocyanins using UV light [108]. Agustin, Safitri, and Fatchiyah, F. (2021) employed the same TLC plate and UV light with n-butanol, AA, and water (3:1:1 *v*/*v/v*) to monitor 4 anthocyanins in red sorghum [109]. Furthermore, they used UV-Vis to determine the Total Anthocyanin Content. Wulandari et al. (2023) also used the same TLC plate and UV light for the chemical profiling of anthocyanin content in four basil cultivars [110]. The research compares TLC results with 1H NMR data, revealing comparable separation power. HPTLC’s potential for chemical fingerprinting and preparative purposes is demonstrated, highlighting its suitability for identifying metabolites in mixture analysis. The study successfully isolates and identifies marker compounds, emphasizing HPTLC’s role in providing efficient and reliable separation for metabolomics studies and chemical profiling of natural products.

Finally, Matus-Castillo et al. (2022) used TLC to monitor the extraction of glycosylated anthocyanins from the shell of radish for use as natural food colorants with high stability [83]. They monitored 14 fractions using Silica gel 60 ADAMANT TLC plate (20 × 20 cm) from Merck, and butanol/HCl/water (6:1:3 *v*/*v/v*) as the mobile phase with UV light. This research highlights the potential of radish-derived anthocyanins for both antioxidant activity and food coloring applications [111].

**Table 3:** Thin-layer Chromatography instrumental features for anthocyanin determination (2020–present).

Sample	TLC Plate	Developing Solvent	Detector	Reference
Red sorghum	Silica gel 60 F254 (20 × 10 cm; 100–200 µm) Merck	EtOAc/Water/FA (85:8:6 *v*/*v*)	UV light	[108]
Pigmented rice	Silica gel 60 F254 (20 × 10 cm; 100–200 µm) Merck	n-butanol/AA/water (3:1:1 *v*/*v*)	UV light	[109]
Basil	Silica gel 60 F254 (20 × 10 cm; 100–200 µm) Merck	EtOAc /FA/AA/Water (100:11:11:27 *v*/*v*)	UV light	[110]
Radish	Silica gel 60 ADAMANT (20 × 20 cm; 250 µm) Merck	Butanol/HCl/water (6:1:3 *v*/*v*)	UV light	[111]

AA, acetic acid; FA, formic acid; EtOAC, ethyl acetate.

## 4. Topical Anthocyanin Analysis

An increasing demand for quality assurance in food production requires sophisticated analytical methods for objective quality control. Traditional analytical methods are precise, but destructive, labor-intensive, time-consuming, and costly. Topical techniques, such as Near-infrared (NIR) Spectroscopy, Fourier Transform Infrared (FTIR) Spectroscopy, and Raman spectroscopy, offer a straightforward, rapid, and cost-effective alternative, and do not require reagents [31,112,113]. NIRS can be used for quality control and assurance in the food industry. It allows for the rapid assessment of various parameters, such as moisture content, fat content, protein content, and sugar content [114], and acidity, antioxidant content, activity, and micronutrient content such as anthocyanins in food products [115]. The absorption IR of any wavelength by a material induces molecular vibrations. There are differences in light energy between the three regions that lead to varying absorptions from different molecules and bonds and induce different types of vibrations. The least energetic region of light is far infrared (FIR), which is absorbed by heavy atoms, such as some inorganic and organometallic substances, while the mid-infrared region (MIR) is better for organic chemical analyses. The near-infrared region (NIR) is the most energetic infrared region. NIR broad peaks could not be directly assigned to specific chemical compounds or interpreted in a straightforward manner as in MIR spectra. Therefore, Fourier Transform (FT), which is widely used in MIR spectroscopy, has recently gained high popularity in the NIR range as well. FT technology has advantages such as high SNR (Signal-to-Noise Ratio), high light outputs due to the absence of slits, fast measurements, instrumental simplicity, and high resolution and accuracy [116]. The disadvantage of IR spectroscopy may be the relatively low penetration depth of infrared radiation into the sample, which may provide information not representative of the sample’s entire volume, especially for a non-homogeneous sample. However, NIR radiation can generally penetrate a sample much further than MIR radiation. The NIR region can penetrate several millimeters into a sample, whereas the penetration power of the MIR region is only in micrometers. The difference in the penetration depth of NIR and MIR may affect the accuracy of the qualitative and quantitative analyses of chemicals [14].

### 4.1. Innovative Spectroscopic Techniques for Food Quality Assurance

IR operates in the infrared region of the electromagnetic spectrum, between 780 nm and 1000 μm; however, the most common in food evaluation is Near-infrared (NIR) Spectroscopy, which is based on the absorption of electromagnetic radiation at wavelengths in the range of 780–2500 nm [29,113,117]. Using FTNIR spectroscopy generally obtains a lower information power than FTIR spectroscopy, which is caused by the absorptions of the functional groups being less intense than the corresponding absorptions of the same functional groups in the all-infrared regions. However, the absorptions of –OH (carbohydrate, protein, and lipid) and –NH (protein) groups are better resolved in NIR than those in the IR region, and the hydrogen bond interactions cause larger band shifts than those observed in the IR region, making their interpretation easier. This makes FTNIR spectroscopy reliable for studies as well because it provides complementary information to that obtained by FTIR [118]. NIR spectroscopy is based on overtones and combinations of fundamental vibrations from the hydrogenous bonds, including numerous C–H, O–H, S–H, and N–H [114]. There are overtones in the NIR region, which means that single-bond absorption peaks appear multiple times throughout the NIR spectrum, with different attenuation levels [29]. Water has a strong infrared absorption that can overshadow other specific peaks. Therefore, Infrared Spectroscopy cannot be applied to food with high moisture content. Whereas, Raman spectroscopy provides a variety of chemical and structural molecular information with good chemical identification. Moreover, water does not have a characteristic Raman shift, so water in the sample is not an obstacle to this method [31].

In addition to identifying the functional groups of chemical compounds and their characteristics, FTNIR spectrometry can be used for routine screening quantitative measurements using predictive models. For this purpose, it is necessary to develop a measurement calibration that uses the relationship between sample spectra and the value of the content/parameter measured by the reference method. In order to be able to test the anthocyanin content in a sample using NIR spectrometry, a prior reference analysis using the HPLC technique is necessary. Chemometric processing is used to create a predictive model for a given parameter. The NIR spectrometry is an analytical technique that reached sample development with the technological revolution, by coupling the NIR spectra with chemometrics [119].

During spectral collection, systemic noise due to light scattering, particle size changes, and instrumental drift can affect FT-NIR and FT-IR spectral data. The FT-NIR and FT-IR spectral data are treated using several preprocessing methods: normalization, multiple scatter correction (MSC), and Savitzky–Golay smoothing (first and second derivatives). The standard normal variable (SNV) is used to correct for changes in spectral information caused by scattering and particle size differences in the sample. To obtain more accurate information from spectroscopic measurements, the raw spectra should be corrected using preprocessing techniques, and NIR calibrations can be performed with multivariate regression (e.g., partial least squares—PLS, Principal Components Regression—PCR) [14].

In the research of Beltrame et al. (2021), a very high correlation coefficient (0.9699) of total anthocyanins in grape juice was obtained between the values from the reference analysis and the estimated one developed by the predictive model using portable Near-infrared (NIR) Spectroscopy [119]. Whereas Eguchi, Sawai, and Sato (2011) developed a calibration for anthocyanin content in corn cob, kernel, and leaf stems for measurement using a NIR spectroscope. They obtained a high correlation coefficient (≥0.81) of the values from the reference analysis and the calibration model [120].

In another study, Miramont et al. (2019) used FTIR spectroscopy and chemometrics to develop partial least squares (PLS) models to predict the concentrations of various anthocyanins in red wine during fermentation. The concentration of molecular anthocyanins was determined by High-performance Liquid Chromatography with ultraviolet–visible detection, and the ratio of monomeric anthocyanins to polymeric anthocyanins was determined using the Adams–Harbertson assay. These authors obtained a high determination coefficient (R^2^) for both calibration and cross-validation exceeding 0.8. Rapid anthocyanin monitoring by FTIR spectroscopy can be helpful for monitoring changes in these compounds over time during winemaking [121].

### 4.2. IR Characterization and Identification of Anthocyanins

Anthocyanins, in addition to carotenoids, chlorophylls, and betalains, are the most common natural pigments of plants. The anthocyanin compounds are glycosylated polyhydroxy or polymethoxy derivatives of 2-phenylbenzopyrilium with two benzyl rings or flavylium salts. In the study of the characteristics of anthocyanins in elderberry performed using NIR spectroscopy, Stuppner et al. (2020) noted 5 main peaks: 8600 cm^−1^ (C–H stretching second overtone), 8328 cm^−1^ (C–H stretching second overtone), 6900 cm^−1^ (O–H stretching and N–H asymmetric stretching first overtone), 5620 cm^−1^ (C–H symmetric stretching first overtone), and 5188 cm^−1^ (O–H stretching and deformation combination) [122].

Research by Dai et al. (2022) showed that extracts of different origins have different anthocyanin compositions [123]. Using surface-enhanced Raman spectroscopy, Dai et al. (2022) confirmed that although the anthocyanin extracts have different chemical compositions, they share some typical specific peaks. Such information can be used to identify anthocyanin. The presence of ring stretching vibration modes of the A and B rings gives peaks at ~1330, 1370, 1530, 1570, 1600, and 1630 cm^−1^ [124].

### 4.3. Advanced Applications and Future Perspectives

The intense peaks at ~1600 cm^−1^ are related explicitly to the flavylium cationic form of the anthocyanins. Moreover, other strong peaks at wavenumber ~1330 cm^−1^ are attributed to the bond stretching modes between the rings and are closely related to the π-electron interaction between the B ring and the rest of the molecule [124]. At wave numbers ~1080 and 1240 cm^−1^, stretching of the C=O bond is revealed, while the peak at ~1190 cm^−1^ is the result of bending of the hydroxyl groups. Moreover, some in-plane and out-of-plane bending modes of the framework can be recognized at ~420, 480, 630, and 640 cm^−1^.

The future of vibrational spectroscopy in food quality assurance promises further advancements in non-destructive testing, with ongoing research aimed at enhancing the sensitivity, specificity, and portability of these techniques. The integration of machine learning and artificial intelligence could revolutionize the interpretation of complex spectral data, enabling real-time, on-site analysis. This evolution underscores the potential of NIR and Raman spectroscopy to meet the increasing demands for quality and safety in the food industry, ensuring the reliability and healthfulness of food products worldwide.

In summary, the complexity of NIR spectra resulting from the large overlap of many bands makes it impossible to identify contributions from the absorption bands of the analyzed anthocyanin compounds in the spectrum of each sample, mainly due to the high moisture content. However, thanks to Raman spectroscopy, a more comprehensive molecular characterization of anthocyanins is possible.

## 5. Challenges and Future Directions

### 5.1. Overview of Current Challenges in Both Quantitative and Topical Methods

Anthocyanins are a large group of plant pigments classified as so-called natural non-nutritive substances of plant origin soluble in water and responsible for their red coloration. These pigments are found in flowers, fruits, leaves, stems, and less often in roots and wood. In cells, they are found in vacuoles, in the form of granules of various sizes, while cell walls and pulp tissues do not contain anthocyanins [125]. Many difficulties may be encountered when determining anthocyanins, often related to sample preparation before analysis. The traditional approach for anthocyanins determination is wet chemical methods that involve pigment extraction in organic solvents (e.g., acetone, methanol, and ethanol) for subsequent spectrophotometric measurements. However, laboratory procedures are laborious, time-consuming, and destructive to the plants [126]. The determination of anthocyanins in plants can be carried out using classical (spectrophotometric) or modern methods—HPLC combined with various types of mass spectrometers or NMR apparatus. However, spectrophotometric methods are insufficient to accurately characterize individual anthocyanins present in plant materials. An inexact correlation between absorbance and actual anthocyanin concentration may be obtained. Gitelson et al. (2009) showed that there are alternatives to wet chemical methods such as reflectance and/or absorption spectroscopy, which are efficient and non-destructive. They developed an index for non-destructive estimation of anthocyanins based on the optical properties of leaves and spectral features of anthocyanins [126].

The latest methods for assessing the anthocyanin content in food products focus on the application of spectroscopic techniques (fluorescence, Raman, nuclear magnetic resonance spectroscopy, Fourier Transform Infrared Spectroscopy, and Near-infrared Spectroscopy), hyperspectral imaging, chemometric-based machine learning, and artificial intelligence applications. The use of topical techniques offers a number of advantages such as chemical-free evaluation, minimal and uninterrupted sample processing, and very rapid and relatively accurate determination of multiple physicochemical properties [17]. Martinez-Sandoval et al. (2016) showed the potential of near-infrared hyperspectral imaging for the screening of anthocyanins in grapes as a fast, reasonably inexpensive, and non-destructive method [127]. Li et al. (2023) described a rapid and high-precision method to detect and visualize anthocyanin content in mulberry fruit. Their results allow us to conclude that hyperspectral imaging visualizes changes in anthocyanin content in fruits at different maturity stages [128]. Yoshioka et al. (2013) investigated the effectiveness of image analysis in estimating anthocyanins content and other fluorescent phenolic compounds that existed by UV rays in strawberry fruit [129]. These results indicate that image analysis can allow for the selection of genotypes rich in anthocyanins. Rapid image analysis can replace time-consuming laboratory work and expensive laboratory equipment. Other authors pointed out the possibility of using FT-NIR and FT-NIR spectroscopic techniques as rapid and non-destructive methods to predict Total Anthocyanin Content and major individual anthocyanins in soybean seed [14].

The use of the above topical methods to estimate the content of anthocyanins in agri-food products requires the initial use of, for example, the HPLC method to determine reference values of anthocyanin concentrations for these products.

### 5.2. Potential Advancements and Innovations in the Field of Anthocyanins Analysis

Although the methods of rapid spectroscopy and image analysis are inexpensive and non-destructive, it should be remembered that they must be properly validated using extractive quantitative analysis.

Nowadays, there is a growing demand for rapid assessment of food quality and safety. There is a need to develop non-invasive technologies for the assessment of food and the content of individual compounds. Future works can be focused on minimizing the disadvantages of the methods described in this work. A combination of different methods could be tried to improve and accurately estimate the anthocyanin content of foods. It seems important to use cheap and easy-to-use techniques and to use simple chemometric algorithms for data interpretation. The use of chemometric artificial intelligence in food analysis seems to be very interesting for the future.

## 6. Conclusions

The analytical landscape for anthocyanin quantification and characterization in food science is diverse, encompassing both time-honored and emerging methodologies, each with its unique advantages and inherent limitations. High-performance Liquid Chromatography (HPLC), with its reliance on silica-based reverse-phase columns, such as C18 and phenyl, remains a gold standard for detailed anthocyanin analysis. The evolution towards Ultra-high-performance Liquid Chromatography (UHPLC) and the advent of novel packing technologies like core–shell columns have significantly enhanced the efficiency, resolution, and speed of these analyses, making them indispensable in the precise quantification and identification of anthocyanins.

Thin-layer Chromatography (TLC), while less sophisticated, serves as an essential preliminary tool for the isolation verification and basic profiling of anthocyanins. Despite its simplicity, the integration of TLC with innovative detection methods, such as smartphone technology, has opened new avenues for its application in quality control, particularly in resource-constrained settings.

The choice of detection techniques—ranging from UV detectors to Mass Spectrometry (MS) and High-resolution Mass Spectrometry (HRMS)—is dictated by the specific objectives of the analysis. UV detectors, including a photodiode array (PDA) and a diode array detector (DAD), provide valuable insights into anthocyanin types during profiling phases. Meanwhile, MS, especially HRMS, is favored for its unparalleled precision in distinguishing between structurally similar anthocyanin molecules, thus offering a comprehensive profiling capability.

Spectroscopic methods, such as Near-infrared (NIR) spectroscopy, Fourier Transform Infrared (FTIR) spectroscopy, and Raman spectroscopy, have gained prominence as cost-effective, rapid, and straightforward alternatives for anthocyanin analysis and food quality control. Their non-invasiveness, minimal preparation requirements, and the absence of reagent use align perfectly with the needs of on-site analysis, marking a significant shift towards more accessible food quality assurance practices.

However, a critical examination of these methodologies reveals that each comes with specific trade-offs. HPLC and UHPLC, despite their analytical robustness, may present barriers in terms of operational complexity and cost, potentially limiting their accessibility. TLC’s utility, on the other hand, is hampered by its lower resolution and quantitative analysis challenges. Spectroscopic methods, while promising for rapid screening, may not achieve the same level of specificity or sensitivity as chromatographic or mass spectrometric techniques. Addressing these limitations calls for a multidisciplinary approach, leveraging the strengths of various methods through hybrid analytical strategies. Future research should thus focus on refining these techniques, exploring innovative detection technologies, and developing integrated systems that offer comprehensive, accurate, and efficient analysis of anthocyanins, ultimately enhancing the quality assurance processes within the food industry.

In the study and analysis of anthocyanins within food products, researchers often find themselves at a crossroads between employing quantitative analysis and topical analysis methodologies. Quantitative analysis, which is heralded for its accuracy and selectivity, offers a deep dive into the precise measurement of anthocyanin concentration, allowing for a detailed understanding of its presence and variations in food. However, this method is often time-consuming and expensive, making it a less viable option for studies requiring rapid or broad-spectrum insights. On the other hand, topical analysis stands out for its rapid and cost-effective nature, providing a qualitative overview that, while less precise, enables a broader survey of anthocyanin presence across various samples. The qualitative nature of topical analysis, however, means it lacks the specificity and selectivity that quantitative methods offer. This dichotomy underscores the necessity of calibrating these approaches against each other, balancing the need for detailed, accurate data with the practicalities of time and budget constraints. By integrating both methodologies, researchers can harness the strengths of each, ensuring a comprehensive analysis that accommodates both the depth of quantitative insights and the breadth of topical observations, thereby offering a more nuanced understanding of anthocyanins in food.
